# APIS: accurate prediction of hot spots in protein interfaces by combining protrusion index with solvent accessibility

**DOI:** 10.1186/1471-2105-11-174

**Published:** 2010-04-08

**Authors:** Jun-Feng Xia, Xing-Ming Zhao, Jiangning Song, De-Shuang Huang

**Affiliations:** 1 Intelligent Computing Laboratory, Hefei Institute of Intelligent Machines, Chinese Academy of Sciences, P.O. Box 1130, Hefei, Anhui 230031, China; 2 School of Life Science, University of Science and Technology of China, Hefei, Anhui 230027, China; 3 Institute of Systems Biology, Shanghai University, Shanghai, 200444, China; 4 Bioinformatics Center, Institute for Chemical Research, Kyoto University, Uji, Kyoto 611-0011, Japan; 5 Department of Biochemistry and Molecular Biology, Monash University, Melbourne, VIC 3800, Australia

## Abstract

**Background:**

It is well known that most of the binding free energy of protein interaction is contributed by a few key hot spot residues. These residues are crucial for understanding the function of proteins and studying their interactions. Experimental hot spots detection methods such as alanine scanning mutagenesis are not applicable on a large scale since they are time consuming and expensive. Therefore, reliable and efficient computational methods for identifying hot spots are greatly desired and urgently required.

**Results:**

In this work, we introduce an efficient approach that uses support vector machine (SVM) to predict hot spot residues in protein interfaces. We systematically investigate a wide variety of 62 features from a combination of protein sequence and structure information. Then, to remove redundant and irrelevant features and improve the prediction performance, feature selection is employed using the F-score method. Based on the selected features, nine individual-feature based predictors are developed to identify hot spots using SVMs. Furthermore, a new ensemble classifier, namely APIS (A combined model based on Protrusion Index and Solvent accessibility), is developed to further improve the prediction accuracy. The results on two benchmark datasets, ASEdb and BID, show that this proposed method yields significantly better prediction accuracy than those previously published in the literature. In addition, we also demonstrate the predictive power of our proposed method by modelling two protein complexes: the calmodulin/myosin light chain kinase complex and the heat shock locus gene products U and V complex, which indicate that our method can identify more hot spots in these two complexes compared with other state-of-the-art methods.

**Conclusion:**

We have developed an accurate prediction model for hot spot residues, given the structure of a protein complex. A major contribution of this study is to propose several new features based on the protrusion index of amino acid residues, which has been shown to significantly improve the prediction performance of hot spots. Moreover, we identify a compact and useful feature subset that has an important implication for identifying hot spot residues. Our results indicate that these features are more effective than the conventional evolutionary conservation, pairwise residue potentials and other traditional features considered previously, and that the combination of our and traditional features may support the creation of a discriminative feature set for efficient prediction of hot spot residues. The data and source code are available on web site http://home.ustc.edu.cn/~jfxia/hotspot.html.

## Background

Protein-protein interactions play a key role in cellular function and form the backbone of most biological processes [1-3]. Although the principles governing protein interactions are not fully understood, it is well known that most of the binding energy in an interaction is contributed by a small portion of the total number of amino acids [4,5]. These amino acids are termed as hot spots that appear to be clustered in tightly packed regions in the center of protein interfaces, and are observed to be crucial for preserving protein function and maintaining the stability of protein association [5-8]. A popular systematic experimental technique for identifying hot spots is through site-directed mutagenesis like alanine scanning [9], which aims to evaluate the change in the binding energy resulting from the mutations of protein side-chains to alanine within a protein interface. A database collecting such experimental hot spots is Alanine Scanning Energetics database (ASEdb) [10]. Another database, i.e., binding interface database (BID), also contains experimentally verified hot spots in protein-protein binding interfaces extracted from the literature [11].

Due to the crucial role played by hot spots, their characteristics have been extensively studied. Several works have disclosed that the amino acid compositions are different between hot spot and non-hot spot regions [6]. Bogan and Thorn reported that hot spots are enriched in Tyr, Trp and Arg due to their size and conformation. They also found that hot spots are surrounded by energetically less important residues that shape like an O-ring to occlude bulk water molecules from the hot spots [12,13].

On the other hand, Leu, Ser, Thr and Val residues [5,6] are disfavored and essentially absent in hot spots despite their importance for protein structures. Analysis of various complexes has also shown that Asn and Asp are more prevalent in hot spots than Gln and Glu [5,6], which might be due to the differences in side-chain conformational entropy.

Furthermore, some studies indicate that the hot spots are more conserved than non-hot spots [14,15]. Ma *et al*. analyzed residue conservation in ten protein families and found that hot spot residues are statistically correlated with structurally conserved residues [16]. Another study [17] illustrated that hot spots from different monomers prefer to interact. The correlation between these couplings and structural conservation was found to be remarkable [18]. Keskin *et al*. [19] found that there is a strong correspondence between experimentally identified hot spots and structurally conserved residues, which can be explained by the observation that the hot spots are located within densely packed regions. They also found that the hot spots are surrounded by residues that are moderately conserved. It has also been shown that hot spots are related to central interface resides, which are conserved in sequence alignments and are not exposed to the solvent in protein complex [16].

Based on the studies on the characteristics of hot spots, a number of computational methods have been developed to predict and identify hot spot residues from interface residues. Generally speaking, these methods can be split into two groups: energy-based methods and feature-based methods. Some energy-based methods, such as computational alanine scanning [20], use a free energy function to calculate the effects of alanine mutations on the binding free energy of a protein-protein complex. Molecular dynamics simulations [21,22] can also be used to estimate the free energy of association. Although these methods give good predictive results, they are not applicable in large scale hot spot predictions due to the high computational cost and the difficulty in operation. On the other hand, the feature-based methods try to discriminate hot spots from the rest of the interface residues by using sequence, structure or a combination of both structure and sequence information. Ofran and Rost [23] used a neural network, based on local sequence environment and evolutionary profile of residues, to identify hot spots. Their method can directly predict hot spot residues from protein primary sequences and suggests that the commonalities of hot spots have been imprinted clearly onto amino acid sequences. Darnell *et al*. [24,25] introduced two decision tree approaches to predict hot spots based on shape-related features and biochemical contact features, respectively. A combination of these two models using a simple OR rule led to better prediction accuracy than computational alanine scanning. Other feature-based methods include those from Guney *et al*. [26] that identify hot spots using solvent accessible surface areas and residue conservation, and a similar one from Tuncbag *et al*. [27] that present an empirical formula to determine hot spots by combining solvent accessible surface areas and statistical pairwise residue potentials. In a more recent work, Cho *et al*. [28] applied a support vector machine (SVM) to predict hot spots with features extracted from sequence, structure and molecular interaction information. Lise *et al*. [29] also employed SVMs as classifiers with input features extracted from the basic energetic terms that contribute to hot spot interaction.

Although current feature-based methods achieve relative success for identifying hot spots in protein interfaces, they are still at the primary stage. Up to now, the biological properties that are responsible for hot spots have not been fully understood. Consequently, the features previously identified as being correlated with hot spots are still insufficient. In this paper, we present a new efficient feature-based method to identify hot spots in protein interfaces. Initially, we extracted a wide variety of features from a combination of protein sequence and structure information. We then performed feature selection to remove noisy and irrelevant features, and thus improved the performance of the classifier. After extensive feature selection, nine individual-feature based predictors were developed to identify hot spots using support vector machines (SVMs). Finally, we employed an ensemble classifier approach, which further improved prediction accuracies of hot spots. To demonstrate its effectiveness, the proposed method was applied to both the ASEdb and BID benchmark datasets. Empirical studies show that our method can yield significantly better prediction accuracy than those previously published in the literature.

## Methods

### Datasets

#### Training Set

The training data set used in this study was extracted from a set of 17 protein-protein complexes defined by Cho *et al*. [28]. It is composed of interface residues experimentally mutated to alanine which have reported free energy of binding (ΔΔG) from the ASEdb database [10] and the published data of Kortemme and Baker [20]. The redundancy in this data set was further eliminated by using the CATH query system with the sequence identity less than 35% and the SSAP score less than or equal to 80. We also removed protein chains for which we could not obtain the corresponding Consurf-DB files [30] from the original data set. A hot spot residue is defined as an interface residue in the data set if its corresponding binding free energy is higher or equal to 2.0 kcal/mol. The interface residue with binding free energy less than 0.4 kcal/mol is considered as non-hot spot, as described by Tuncbag *et al*. [27]. Other interface residues with binding free energy between 0.4 and 2.0 are excluded from the training set in order to better discriminate. According to the above definitions, we obtained 154 interface residues, of which 62 residues are hot spots and 92 residues are non-hot spots, as shown in Table 1 and Additional file 1.

**Table 1:** Training set of protein structures

PDB	First molecule	Second molecule
1a4y	Angiogenin	Ribonuclease Inhibitor
1a22	Human growth hormone	Human growth hormone binding protein
1ahw	Immunoglobulin Fab 5G9	Tissue factor
1brs	Barnase	Barstar
1bxi	Colicin E9 Immunity Im9	Colicin E9 DNase
1cbw	BPTI Trypsin inhibitor	Chymotrypsin
1dan	Blood coagulation factor VIIA	Tissue factor
1dvf	Idiotopic antibody FV D1.3	Anti-idiotopic antibody FV E5.2
1fc2	Fc fragment	Fragment B of protein A
1fcc	Fc (IGG1)	Protein G
1gc1	Envelope protein GP120	CD4
1jrh	Antibody A6	Interferon-gamma receptor
1vfb	Mouse monoclonal antibody D1.3	Hen egg lysozyme
2ptc	BPTI	Trypsin
3hfm	Hen Egg Lysozyme	lg FAB fragment HyHEL-10

#### Independent test set

An independent test set was extracted from the BID database [11] to further assess the performance of our proposed method. In BID, the relative disruptive effect of the mutation is listed as either 'strong', 'intermediate', 'weak' or 'insignificant'. In our study, hot spot residues are labeled as the ones with 'strong' mutations and others are regarded as non-hot spots. Note that we used exactly the same dataset as the one used in Cho *et al*. [28] for the purpose of comparing our method with theirs, because their method is currently the state-of-the-art in the field of hot spot prediction based on protein structures.

### Feature representation

To build a predictor that can best distinguish hot spot residues from non-hot spots, we performed an extensive search so as to derive, optimize and evaluate features based on the sequence and structure characteristics of protein binding sites. These features (see Additional file 2) can be roughly divided into three groups: (i) Physicochemical features; (ii) Features based on protein tertiary structures; and (iii) Residue-residue pairing preferences at the interface, residue evolutionary conservation scores and temperature factors.

#### Physicochemical features

Physicochemical features of an amino acid residue were described by ten values: number of atoms, number of electrostatic charge, number of potential hydrogen bonds, hydrophobicity, hydrophilicity, propensity, isoelectric point, mass, expected number of contacts within 14 Å sphere, and electron-ion interaction potential. Previous works [31-34] suggest that these ten values correlate well with the interface properties of a protein. The values of the ten physicochemical properties for each amino acid can be found in Additional file 3. These values were only related to the amino acid types and did not contain any structural information.

#### Features based on protein structure

Structure-based features include accessible surface area (ASA) [35], relative ASA (RASA) [36], depth index (DI) [37,38], and protrusion index (PI) [39]. For ASA and RASA, we obtained five residue attributes: total (sum of all atom values), backbone (sum of all backbone atom values), side-chain (sum of all side-chain atom values), polar (sum of all oxygen, nitrogen atom values) and non-polar (sum of all carbon atom values). For DI and PI, we used four residue attributes: total mean (mean value of all atom values), side-chain mean (mean value of all side-chain atom values), maximum (highest of all atom values) and minimum (lowest of all atom values). The structure information in both isolated monomer (unbound) and complex (bound) form was calculated by PSAIA [36,40]. In addition, the relative changes in ASA, DI and PI between the complex and monomer state of the residues were also calculated as follows:(1)

As a result, we obtained 49 structural features. More details can be found in the Additional file 2

#### Features derived from residue-residue pairing preferences at the interface, residue evolutionary rate and temperature factor

It has been shown that the pairwise residue potentials of the interface residues may be useful for improving the prediction of hot spots. For example, Tuncbag *et al*. [27] used knowledge-based solvent mediated inter-residue potentials [41] and solvent accessibility to identify computational hot spots. They found that pairwise potential is a major discriminative feature in hot spot prediction. Here, we obtained features derived from protein interface potentials according to their method. For more details about the implementation of their algorithm, please refer to the original paper [27,41].

Temperature factor is a measure of atomic thermal motion and disorder. It was suggested that interface residues have lower temperature factors than the protein exterior, which generally reflects the lesser flexibility of the interfacial regions [42]. As a result, it has been used to improve the prediction of protein-protein interaction sites. Here, the temperature factor of Cα atom was used to represent the flexibility of each residue and normalized as follows:(4)

where *B*_*r *_represents the temperature factor of residue *r*,  and σ(*B*) are the mean and standard deviation of the temperature factors of the chosen chain.

Residue evolutionary rate is a conservation score to quantify the evolutionary information. The residue conservation score represents a natural indicator to compare the conservation level of any residue in a protein sequence. Thus, the lower the value, the more conserved the corresponding residue of the protein. In our experiment, the evolutionary rate for each residue was obtained using the Rate4Site algorithm [43], which is implemented in the ConSurf-DB server [30].

### Feature selection

Feature selection, more precisely feature subset selection, aims at finding *p *features out of the original *d *ones according to a selection criterion. Note that it is different from feature extraction, where a *d*-dimensional feature vector is projected to a *p*-dimensional subspace (e.g. principal component analysis). Feature selection is an important step in designing classifiers. With feature selection, we can readily remove redundant and irrelevant features to further improve the performance of a classifier. In this work, 62 multifaceted features were generated as described before. It is apparent that the models built based on these large sets of features would overfit the training data. Therefore feature selection needs to be performed to generate robust and general prediction models. In the present work, feature selection was performed using the F-score [33], which assesses the discriminatory power of each individual feature. The F-score was calculated as:(5)

where  and  are the averages of the non-hot spots and hot spots, and σ_*n *_and σ_*hi *_are the corresponding standard deviations, respectively. In other words, the F-score measures the separation of the means for two populations (hot spots and non-hot spots) in terms of their variances, and it is very closely related to the F-statistics, which is commonly used to evaluate the separation of the means for two random variables.

### Model construction

The classification model for predicting hot spots was based on SVM [44], which is a class of effective supervised learning methods that demonstrate high prediction accuracy whilst efficiently avoiding the overfitting problem [45]. In this study, the software LIBSVM [46] was employed and the radial basis kernel function was selected to build the SVM models [38,45]. As discussed by Tuncbag *et al*. [27], residues in the same or homologous binding interfaces generally can not be expected to be independent. However, they found that the results of 10-fold cross-validation and the 'leave one protein complex out' cross-validation show similar results. Therefore, the SVM models were created with a set of default parameters and executed with 10-fold cross-validation for the training set. To further validate our models, the performance was evaluated using the independent test set from the BID.

### Performance evaluation

To assess the performance of classification methods, we adopted a number of commonly used measures: specificity, recall, precision, accuracy and F1 score. These evaluation measures were defined as follows:(6)

where TP, FP, TN and FN represent true positive (correctly predicted hot spot residue), false positive (non-hot spot residue incorrectly predicted as hot spot), true negative (correctly predicted non-hot spot residue) and false negative (hot spot residue incorrectly predicted as non-hot spot), respectively.

## Results and Discussion

The results in this section are presented in the following order. First, we constructed a variety of 62 features from a combination of protein sequence and structure information, and identified the best nine top-ranking features for predicting hot spots. Then, we compared the prediction performance of different machine learning approaches, and found that SVM is the most accurate predictor of binding hot spots. Finally, we combined the individual-feature based SVM predictors, and demonstrated that the ensemble classifier of these single-feature SVMs can significantly improve the predicted hot spot accuracy when compared with other methods based on the independent test set.

### Assessment of feature importance

**Figure 1:**
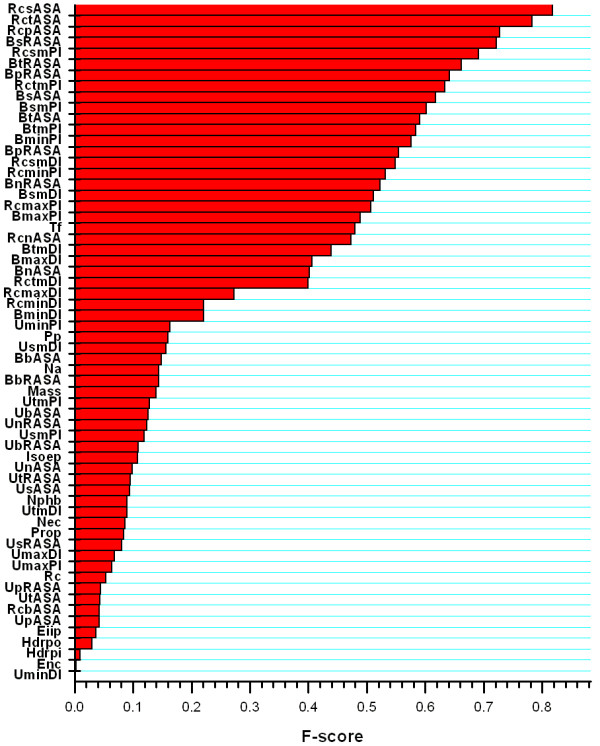
**Feature importance**. This figure presents the importance of 62 particular features and their contribution to the discriminative quality (in descending order) as measured by F-score. The meanings of the feature symbols are described in Additional file 2.

In previous studies, many features have been adopted to improve the predictions of hot spot residues such as accessible surface area (ASA), residue conservation, physicochemical features, and computational alanine scanning. In light of these studies, we first designed and quantified a total of 62 multifaceted features from a combination of protein sequence and structure information. These features include: ten physicochemical characteristics, residue pairwise potential (Pp) at the interface, residue conservation (Rc), temperature factor (Tf), and 49 structure features based on ASA, depth index (DI) and protrusion index (PI). Since one of our goals is to find a more discriminative and smaller feature set for hot spot prediction, we evaluated individual features in terms of their discriminative power, as measured by the F-score which was defined in the Feature Selection Section. The F-score pinpoints the difference in multifaceted features between hot spots and non-hot spots. The training set was used to compute the F-scores. Figure 1 shows the importance of 62 features and their contribution to the discriminative quality (in descending order). As can be seen, the most important features are those based on protein structure information, such as the ASA-based features. Consistent with earlier finding [47], among the features based on structure information, there is a drop in the value of F-score when comparing unbound structures with bound structures. This means that the features derived from protein complex can provide better discriminative power than the unbound structures. This finding can be explained by the fact that the protein binding is usually subject to conformational changes. As a result, the structure of the binding site can differ between structures of the same protein with bound or unbound chains. In the bound structure, the relevant side-chains are in conformations that are in contact with another protein chain, enabling the binding pocket more clearly defined than that in the unbound structure. In other words, the bound structure can provide additional important information that is useful for predicting hot spots.

As reported by Cho *et al*. [28], the relative change in ASA upon complexation (RcASA, includes the relative change in total, backbone, side-chain, polar and non-polar ASA upon complexation) shows better discriminative power than the other corresponding ASA-based features. For example, the F-score of relative change in total ASA upon complexation (RctASA) is higher than both the Unbound total ASA (UtASA) and Bound total ASA (BtASA). We also found that the relative changes in DI and PI upon complexation (RcDI and RcPI, include the relative change in total mean, side-chain mean, maximum and minimal DI and PI upon complexation, respectively), are better than the other DI-based and PI-based features in their ability to discriminate hot spots from non-hot spots. In addition, the side-chain information shows the best discriminative power, with the F-scores of 0.82 (relative change in side-chain ASA upon complexation, RcsASA), 0.69 (relative change in side-chain mean PI upon complexation, RcsmPI) and 0.55 (relative change in side-chain mean DI upon complexation, RcsmDI), respectively. The side-chains of amino acids are known to be responsible for shaping different properties of individual amino acids and can thus endow the proteins with unique structural and functional properties. In addition, it is observed that the side-chain atoms constitute about 80% of the interface area of the average protein complex, while the backbone atoms constitute only about 19% [48]. Therefore, the properties of side-chains contribute considerably to the physicochemical properties of proteins. Moreover, protein-protein complexation is determined by inter-atomic interactions between monomers, of which the interactions between side-chain atoms dominate at the interface. It has been shown that interactions between side-chain atoms are prominent among hot spots [17]. These may explain why the side-chain information plays an important role in the discrimination of hot spots from non-hot spots.

Previous work indicated that there is a correspondence between the hot spots and the evolutionarily conserved residues [26]. However, in this study, we observed that the residue conservation (Rc) score is less informative, with the F-score of only 0.0538. This scenario is consistent with the finding of Tuncbag *et al*. [27]. Although hot spot residues are often conserved, many other residues can be evolutionarily conserved as well, due to other structural and functional constraints. Moreover, hot spots are often surrounded by residues that are moderately conserved [23]. Therefore, it is understandable that the conservation score may not be a good discriminative factor [27].

Interestingly, we found that residue pairwise potentials (Pp), which have been previously adopted to enhance the hot spot prediction [27], did not perform well in this study. In addition, it was observed that another similar sequence-based feature, the expected number of contacts within 14 Å sphere (Enc), also appears to be insignificant. Previous works have suggested that residues with relatively low temperature factors are mainly involved in protein binding. Hence, temperature factor is possibly useful for improving the prediction performance of hot spots. As can be seen from Figure 1, the temperature factor (Tf) has the F-score of 0.4793. It is worth mentioning that the differential distributions of the means separated by the average standard deviations will result in an F-score of 0.5, indicating that the temperature factor can only make a minor contribution to distinguishing hot spots from other non-hotspot residues.

### Individual-feature based classifiers

After extensive feature selection, we selected nine best top-ranking structural features with their respective F-scores higher than 0.60: relative change in side-chain ASA upon complexation (RcsASA), relative change in total ASA upon complexation (RctASA), relative change in polar ASA upon complexation (RcpASA), bound side-chain RASA (BsRASA), relative change in side-chain mean PI upon complexation (RcsmPI), bound total RASA (BtRASA), bound polar RASA (BpRASA), relative change in total mean PI upon complexation (RctmPI) and bound side-chain ASA (BsASA). These features belong to the ASA-based and PI-based features. SVM classifiers were then built to discriminate between hot spots and non-hot spots based on these individual features.

The prediction performances of individual feature-based SVM models are illustrated in Table 2, which were evaluated based on 10-fold cross-validation and the independent test set. We want to emphasize that as a robust metric of classifier performance for problems with unbalanced classes, a better F1 score has to exceed the frequency of hot spots observed in the data set that represents the practical baseline of a random predictor. As the training set consists of 62 hot spots and 92 non-hot spot residues, the F1-score for any model should be more than 0.40. For the independent test set with 127 mutated interface residues of which 39 residues are hot spots, the F1-score should be larger than 0.31. As can be seen in Table 2, the recall, precision and F1 scores of all classifiers are respectively higher in ASEdb, but lower in BID. Nevertheless, these classifiers provide significantly better performance than the random models in both ASEdb and BID (Note that ASEdb is the training set while BID is the independent test set). The performance difference of these individual-feature based models on the two distinct datasets possibly indicates the different natures of these two datasets [27]. The hot spots in ASEdb are defined as the residues for which alanine mutation causes a significant increase in the binding energy of at least 2 kcal/mol. However, in BID, instead of using a single threshold, alanine mutation data are divided into 'strong', 'intermediate', 'weak' and 'insignificant' interactions, and only 'strong' interaction strengths would be considered as hot spots. In BID, the classifier based on the RctmPI feature has the highest F1 score (0.62), while the performance of the classifier based on the RcsmPI feature was the second. Although the classifiers based on RctmPI and RcsmPI were not the most effective in identifying hot spots in ASEdb, they were superior to the majority of classifiers based on individual features. These results indicate that RctmPI and RcsmPI play vital roles in identifying hot spots. The protrusion index (PI) quantifies the extent to which a residue protrudes from the surface of a protein. Pintar *et al*. [39] suggested that the identification of protruding, or highly convex regions in proteins is important in the analysis of interfaces in protein-protein complexes. Wu *et al*. [49] also found that the interface residues tend to protrude from the surface. These analyses explain why the classifiers based on PI perform well. In addition, in accordance with some other recent studies, the classifiers based on the conventional ASA features such as RctASA also have high prediction accuracy.

**Table 2:** Prediction performance of individual-feature based SVM models

Feature	Dataset	Specificity	Recall	Precision	Accuracy	F1	TP	TN	FP	FN
RcsASA	Training set	0.79	0.74	0.71	0.77	0.72	46	73	19	16
	Test set	0.66	0.67	0.46	0.66	0.55	26	58	30	13
RctASA	Training set	0.78	0.71	0.69	0.75	0.70	44	72	20	18
	Test set	0.68	0.72	0.50	0.69	0.59	28	60	28	11
RcpASA	Training set	0.78	0.79	0.71	0.79	0.75	49	72	20	13
	Test set	0.70	0.59	0.47	0.67	0.52	23	62	26	16
BsRASA	Training set	0.72	0.79	0.65	0.75	0.72	49	66	26	13
	Test set	0.52	0.72	0.40	0.58	0.51	28	46	42	11
RcsmPI	Training set	0.75	0.81	0.68	0.77	0.74	50	69	23	12
	Test set	0.74	0.69	0.54	0.72	0.61	27	65	23	12
BtRASA	Training set	0.72	0.69	0.62	0.71	0.66	43	66	26	19
	Test set	0.56	0.72	0.42	0.61	0.53	28	49	39	11
BpRASA	Training set	0.62	0.82	0.59	0.70	0.69	51	57	35	11
	Test set	0.53	0.67	0.39	0.57	0.49	26	47	41	13
RctmPI	Training set	0.76	0.73	0.67	0.75	0.70	45	70	22	17
	Test set	0.78	0.67	0.58	0.75	0.62	26	69	19	13
BsASA	Training set	0.61	0.81	0.58	0.69	0.68	50	56	36	12
	Test set	0.61	0.59	0.40	0.61	0.48	23	54	34	16

It was previously shown that a protein-protein interface is generally more solvent accessible and protruding than other parts of a protein's surface [50]. Li *et al*. [51] analyzed the geometrical features of interfacial residues and found that the complemented pockets and protruding residues are enriched in hot spots as the most important geometric features in protein interfaces. By means of expelling water molecules, the two component chains protrude deeply into one another in protein interfaces so that the complementary pockets of one chain bind to their corresponding protruding residues from the partner chain, and eventually, bind to protect each other from the solvent. In conclusion, both the protrusion index (PI) and accessible surface area (ASA) are the important features to distinguish hot spots from non-hot spots (Table 2).

It is well known that SVM is supposed to have more prediction power based on multiple features rather than individual properties. To further explore this possibility, we also tried multi-feature-based SVMs to predict hot spots. We have normalized these features with the mean and standard deviation of the sample set [28] before inputting them into SVM to build the classifiers. Firstly, the two best features, RcsASA and RctASA, were selected to construct a multi-property predictor, and then other features were added one by one to SVM in decreasing order of F-score to construct a series of multi-feature predictors. The prediction results for the multi-feature predictor with different combinations are shown in Additional file 4. As can be seen, the prediction performance of the multi-features predictor increases from 0.56 to 0.60 when the number of properties increases from 2 to 5. A slight decrease in performance from 0.60 to 0.58 is observed when the number of properties increases from 5 to 9. The results indicate that the performance based on multiple features (maximum F1 = 0.60) was lower than the SVM classifiers based on individual features RcsmPI (F1 = 0.61) and RctmPI (F1 = 0.62). One possible reason might be that there exists correlation among these features (Table 3). For example, the correlation coefficients among the majority of the ASA-based features are larger than 0.60 and the correlation coefficient between the feature RctmPI and RcsmPI is larger than 0.90. Therefore, we used the individual-feature based classifiers as our final models to infer hot spot residues in protein interfaces.

**Table 3:** The correlation coefficients among the nine best top-ranking features

Feature	RcsASA	RctASA	RcpASA	BsRASA	RcsmPI	BtRASA	BpRASA	RctmPI	BsASA
RcsASA	1.0000	0.9714	0.7168	-0.8382	0.8582	-0.8454	-0.6826	0.8007	-0.7866
RctASA		1.0000	0.7733	-0.8234	0.8609	-0.8632	-0.7299	0.8357	-0.7931
RcpASA			1.0000	-0.6201	0.6752	-0.6364	-0.6932	0.6770	-0.5972
BsRASA				1.0000	-0.7052	0.9555	0.7933	-0.6724	0.9239
RcsmPI					1.0000	-0.7477	-0.6730	0.9536	-0.6601
BtRASA						1.0000	0.8563	-0.7084	0.8966
BpRASA							1.0000	-0.6640	0.7465
RctmPI								1.0000	-0.6399
BsASA									1.0000

### Comparison of different classifiers based on the same feature set

Darnell *et al*. [24] indicated that the SVM-based model is considerably worse than the decision tree and Bayes Net models. In this study, in order to identify the best machine learning technique suitable for predicting hot spots in protein interfaces, we comprehensively evaluated the performances of SVM, Bayes Net, Naïve Bayes, RBF Network, Decision Tree and Decision Table classifiers. All these algorithms except SVM were implemented using the Weka package [52] with the default parameter configuration. The performance comparison of the RcsASA feature based machine learning classifiers using 10-fold cross-validation and the independent test set is listed in Table 4. It can be seen that SVM outperformed the Decision Tree and Bayes Net in terms of the F1 score based on the ASEdb dataset, with the F1 score increasing by more than 0.12 and 0.10, respectively. Moreover, it also outperformed RBF Network and Decision Table with the F1 score increasing by more than 0.05 and 0.10, respectively. When compared with Naïve Bayes based on the ASEdb dataset, the F1 score of SVM is only 0.02 smaller than that of Naïve Bayes. Nevertheless, when tested on the BID dataset, the F1 score of SVM is much higher than those of Decision Tree, Decision Table and Bayes Net, with ΔF1 score of 0.21, 0.20 and 0.20, respectively. In addition, SVM has at least comparable performance with Naïve Bayes and RBF Network on the BID dataset. It is worth mentioning that we have also tried machine learning methods based on other features and still got the similar performance (data not shown). The main reason for the performance difference of our method and previous studies is probably due to the application of the novel features in our method. All the above findings indicate that SVM gives better predictive performance compared with Bayes Net, Decision Tree and Decision Table. And it also performs comparably to Naïve Bayes and RBF Network.

**Table 4:** Evaluation of the hot spot prediction using different machine learning classifiers based on the RcsASA feature

Classifier	Dataset	Specificity	Recall	Precision	Accuracy	F1	TP	TN	FP	FN
SVM	Training set	0.79	0.74	0.71	0.77	0.72	46	73	19	16
	Test set	0.66	0.67	0.46	0.66	0.55	26	58	30	13
Bayes Net	Training set	0.79	0.56	0.65	0.70	0.60	35	73	19	27
	Test set	0.85	0.28	0.46	0.68	0.35	11	75	13	28
Naïve Bayes	Training set	0.75	0.81	0.68	0.77	0.74	50	69	23	12
	Test set	0.58	0.72	0.43	0.62	0.54	28	51	37	11
RBF Network	Training set	0.85	0.63	0.74	0.76	0.67	39	78	14	23
	Test set	0.76	0.62	0.53	0.72	0.57	24	67	21	15
Decision Tree (J48)	Training set	0.87	0.53	0.73	0.73	0.62	33	80	12	29
	Test set	0.84	0.28	0.44	0.67	0.34	11	74	14	28
Decision Table	Training set	0.79	0.56	0.65	0.70	0.60	35	73	19	27
	Test set	0.85	0.28	0.46	0.68	0.35	11	75	13	28

### Ensemble classifier for hot spot prediction

In this study, different models were further combined by majority voting in order to improve the final prediction performance. We compared the classifier number used for each model and the results on the independent test set are shown in Table 5. When all the individual feature based classifiers are used, the model achieves a performance of 57%, as measured by the F1 score. Using classifiers based on seven individual features with the F1 score more than 0.50 as the base classifiers, we were able to achieve a slightly better accuracy. A large and significant increase in prediction performance was achieved by combining only three individual feature based classifiers with the F1 scores higher than 0.59. From Table 5, we can see that the majority voting rule based model correctly predicts 72% of hot spots, as compared with 67% for the best individual feature RctmPI based model. However, the majority voting model loses some precision and specificity relative to the RctmPI based model, but increases the recall on the other hand. In other words, more positive hot spots are predicted with a slight lower percentage of true negatives. Each single-feature based model can correctly predict a different subset of hot spots. In summary, we conclude that our ensemble model for predicting hot spots achieved a satisfactory performance.

**Table 5:** Evaluation of hot spot prediction using the majority voting method based on the independent test set

Classifier number	Specificity	Recall	Precision	Accuracy	F1	TP	TN	FP	FN
9 (all)	0.67	0.69	0.48	0.68	0.57	27	59	29	12
7 (F1 > 0.50)	0.68	0.69	0.49	0.69	0.58	27	60	28	12
3 (F1 > 0.59)	0.76	0.72	0.57	0.75	0.64	28	67	21	11

### Comparison with other methods

In this section, we further compared our method with other methods. Our final prediction model is called APIS, an acronym of "A combined model based on Protrusion Index and Solvent accessibility". Table 6 summarizes the performance comparison of different methods on the same independent test set. Among these approaches, Robetta [20] and FOLDEF [53] are alanine scanning methods, while KFC [24,25] and MINERVA [28] are knowledge based methods. Both our method and MINERVA showed high success rates in contrast to the other three methods. The F1 scores for our method and MINERVA are 0.64 and 0.52, respectively, while the other methods have F1 scores in the range of 0.34~0.40. Therefore, the MINERVA and our method can effectively distinguish between hot spots and non-hot spots. Our method can correctly predict hot spots from the data set with recall = 0.72 and precision = 0.57. This means that our method can correctly predict 72% of the true hot spots for this data set (recall), and 57% of the predicted hot spots are identified as true hot spots (precision). MINERVA efficiently identified non-hot spots (specificity = 0.90), while it could not correctly identify most hot spots (recall = 0.44). The F1 score of our model is 12 percentage points higher than that of MINERVA (the detailed comparison of the two methods can be found in Additional file 5). From these analyses, we can see that our method gives remarkably better prediction performance in comparison to other available prediction approaches.

**Table 6:** Performance comparison with different methods based on the independent test set

Method	Specificity	Recall	Precision	F1	ΔF1
Robetta	0.87	0.33	0.52	0.40	**
FOLDEF	0.88	0.26	0.48	0.34	-0.06
KFC	0.85	0.31	0.48	0.37	-0.03
MINERVA	**0.90**	0.44	**0.65**	0.52	+0.12
APIS (this work)	0.76	**0.72**	0.57	**0.64**	**+0.24**

The highest value in each column is shown in bold.

Since the method utilized in our experiments are quite similar to the work by Tuncbag *et al*. [27], it is reasonable to compare our APIS method with their method. However, it is not straightforward to make a direct comparison. For example, we note that on the BID-derived dataset of Tuncbag, the reported F1 score of the Robetta method is 0.60, which is substantially higher than that obtained on our BID-derived dataset (F1 = 0.40). Therefore, to further evaluate the robustness of our method, additional experiments were performed (see Additional file 6) based on the BID-derived dataset of Tuncbag. The comparison results are given in Additional file 6: Supplemental Table S8, which clearly shows that the performance of our method outperforms the other methods to a greater extent, especially the recall value. A higher recall generally means a better prediction of the positive classes and it is thus helpful for the identification of hot spot residues in practical applications. At the same time, we want to emphasize that, although APIS achieves this high recall at the expense of some precision compared with Tuncbag's method, the F1 score indicates that an adequate balance is still achieved between the two measures (the detailed comparison of the two methods can be found in Additional file 5).

One point that should be emphasized in evaluating the significance of hot spot residue prediction is the limited availability of experimental data set of alanine mutations. Both the ASEdb and BID datasets are relatively small and obsolete. The paucity of the experimental data available may cast doubts on the effective relevance of the features that are used to improve the prediction. Lise *et al*. [29] pointed out that the BID may be unsuitable for assessing the power of a hot spot prediction method. As a result, there is a need to establish a substantially larger benchmark dataset of hot spots and non-hot spot residues from current literature to draw better conclusions as to what are the major determinants of hot spots and non-hot spots [23,27].

### Case studies

To further illustrate the effectiveness of our approach APIS for identifying hot spot residues, we present two examples that are predicted by APIS, MINERVA and KFC using VMD software [54].

**Figure 2:**
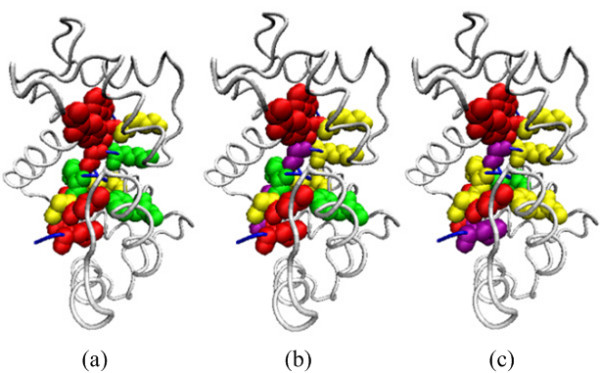
**The visualization of prediction results for chain A (white) and chain E (blue) of protein complex 1CDL using (a) APIS, (b) KFC, and (c) MINERVA**. The following color scheme is used: true positives (known hot spots predicted correctly) in red, true negatives (actual non-hot spots predicted correctly) in yellow, false positives (non-hot spots predicted as hot spots) in green, false negatives (known hot spots not predicted correctly) in purple. In this case, 9 of 12 residues are correctly predicted by our method.

The first example is calmodulin/myosin light chain kinase complex [55]. Calmodulin (CaM, pdbID: 1cdl, chain A) is a calcium-binding protein expressed in all eukaryotic cells [56]. CaM can bind to and mediate a large number of enzymes and other proteins by Ca^2+^. Among the enzymes to be stimulated by the calcium-calmodulin complex are a number of protein kinases such as myosin light chain kinase (MLCK, pdbID: 1cdl, chain E). Experimentally verified hot spot residues in 1cdlAE interface are F92_A, W800_E, G804_E, I810_E, R812_E and L813_E. Moreover, F12_A, F19_A, K799_E, K802_E, R808_E and G811_E are found experimentally to be non-hot spots. As a comparison, our method can correctly predict the whole set of hot spots, while KFC only correctly predicts three hot spots and MINERVA identifies four hot spots (Figure 2, Additional file 7). In addition, our method can also correctly predict three out of the six non-hot spots, which are F12_A, K799_E and G811_E. As a contrast, KFC and MINERVA can identify four non-hot spot residues (F12_A, K799_E, K802_E and G811_E), and five non-hot spot residues (F19_A, K799_E, K802_E, R808_E and G811_E), respectively. Although KFC and MINERVA obtained a higher number of non-hot spots, they can identify fewer hot spots. Altogether, 9 out of the 12 residues can be correctly predicted by APIS.

**Figure 3:**
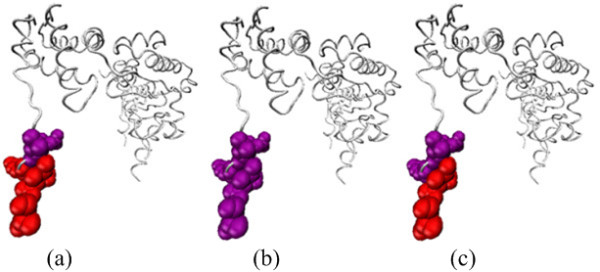
**The visualization of prediction results for chain A (white) of protein complex 1G3I (Chain G not shown) using (a) APIS, (b) KFC, and (c) MINERVA**. Red residues are actual hot spots predicted correctly, purple residues are actual hot spots not predicted correctly.

Another example is heat shock locus gene products U and V (HslUV) complex [57]. As a bacterial homolog of the eukaryotic proteasome, the HslUV complex is composed of the heat shock locus gene products V (HslV) protease and the heat shock locus gene products U (HslU) ATPase. HslU (pdbID: 1g3i, chain A) is a molecular chaperone that facilitates the degradation of target proteins. When HslU binds to its cognate protease HslV (pdbID: 1g3i, chain G), the proteolytic activity of HsIV is enhanced one or two orders of magnitude. HslU has experimentally determined six hot spots in its binding sites to HslV. These hot spots gather locally and form a hot region. Our method can correctly predict four of these six residues to be hot spots (R441, F442, I443 and L444), while KFC predicts all six residues as non-hot spots and MINERVA only identifies F442, I443 and L444 as hot spots correctly (Figure 3, Additional file 7). D438 and L439 are not predicted to be hot spots by all these three methods, suggesting that mutations of these two residues might contribute to protein destabilization.

These prediction results clearly demonstrate that the potential of APIS in identifying more hot spot residues than other methods at the given thresholds, with little compromise in precision (as the F1 scores indicate). In conclusion, it was shown in computational experiments that the proposed method outperforms two other state-of-the-art methods.

## Conclusion

Hot spots are residues comprising only a small fraction of interface residues yet contributing significantly to the binding free energy. In this study, we propose a new efficient method to computationally determine hot spots in the protein interface, given the structure of a protein complex. Both the new features based on the protrusion index and the traditional features based on solvent accessibility of interface residues are used as the input to SVM classifiers. Our analysis implies that solvent occlusion is an indispensable factor to define a hot spot residue, but not sufficient itself. We also show that residue conservation and temperature factor do not have significant effects on hot spot prediction when used as individual features alone. Interestingly, residue-residue pair potentials, which were found to be effective in previous studies, could not significantly improve the prediction of hot spot residues. Our results show that residue occlusions from solvent and protrusion index are the main discriminative features in hot spot prediction. The performance of our approach was firstly evaluated using the 10-fold cross-validation and further validated using an independent test set from the BID dataset. The experimental results show that our APIS approach can provide favourable or at least comparable performance compared with all the previous methods and complement the experimental techniques that were developed to identify hot spots.

Although the final best model is based on solvent accessibility and protrusion index of interface residues, novel characteristic features that better describe the different energetic contributions of the interface residues can be easily incorporated into our prediction system to further improve the prediction performance of hot spots. Researchers who are interested in finding new features of hot spot residues could use the APIS model to characterize the roles of their features. APIS would also benefit from these new features on the other hand. In our future work, we will offer an online web interface through which our APIS approach can be implemented to computationally identify potential hot spots.

## Authors' contributions

JX designed the study, implemented the prediction system, drafted the manuscript and performed the analysis. XZ and JS participated in the design of the study, drafted the manuscript and performed the statistical analysis. DH conceived of the study, and drafted the manuscript. All authors read and approved the final manuscript.

## Supplementary Material

Additional file 1 **Alanine mutated interface residues in the training dataset**. The dataset contains 62 hot spot residues and 92 non-hot spot residues. Click here for file

Additional file 2 **Summary of the features used in this study**. These features can be roughly divided into three groups: (i) physicochemical features; (i) features based on protein tertiary structures; and (iii) residue-residue pairing preferences at the interface, residue evolutionary conservation scores and temperature factors. Click here for file

Additional file 3 **Physicochemical features**. Values of the ten physicochemical features are contained in this file. Click here for file

Additional file 4 **The average prediction results of multi-property SVMs for different number of properties based on independent test set**. The feature RcsASA, RctASA, RcpASA, BsRASA, RcsmPI, BtRASA, BpRASA, RctmPI and BsASA were added one by one to construct a series of multi-property SVMs according to the corresponding F-scores. Click here for file

Additional file 5 **Comparison of methodologies**. The methodological difference between our method and the other two previous methods of Tuncbag *et al*. (2009) and Cho *et al*. (2009). Click here for file

Additional file 6 **Performance based on the Tuncbag *et al*. dataset assembled from BID**. Table S6: Performance of individual-feature based SVM models; Table S7: Evaluation of hot spot prediction using the majority voting method; Table S8: Performance comparison with different methods. Click here for file

Additional file 7 **Performance on the test set (BID)**. Detailed prediction results for the protein structures obtained with our method. Click here for file
