# Supplementary material for: APIS: accurate prediction of hot spots in protein interfaces by combining protrusion index with solvent accessibility
Source: BMC Bioinformatics. 2010 Apr 8;11:174. doi: 10.1186/1471-2105-11-174 (PMC2874803; doi:10.1186/1471-2105-11-174)
Supplement: Additional file 1 — Alanine mutated interface residues in the training dataset. The dataset contains 62 hot spot residues and 92 non-hot spot residues. [file 1471-2105-11-174-S1.DOC]

Table S1 Alanine mutated interface residues in the training set.

| **PDB** | **Chain** | **Residue** | **Sequence** | **ΔΔGobs** |
| --- | --- | --- | --- | --- |
| 1a4y | A | W | 261 | 0.1 |
| 1a4y | A | S | 289 | 0 |
| 1a4y | A | K | 320 | -0.3 |
| 1a4y | A | E | 344 | 0.2 |
| 1a4y | A | Y | 434 | 3.3 |
| 1a4y | A | D | 435 | 3.5 |
| 1a4y | A | R | 457 | -0.2 |
| 1a4y | B | R | 5 | 2.3 |
| 1a4y | B | Q | 12 | 0.3 |
| 1a4y | B | H | 13 | -0.3 |
| 1a4y | B | R | 31 | 0.2 |
| 1a4y | B | N | 68 | 0.2 |
| 1a4y | B | H | 84 | 0.2 |
| 1a4y | B | W | 89 | 0.2 |
| 1a4y | B | E | 108 | -0.3 |
| 1a22 | A | H | 18 | -0.5 |
| 1a22 | A | H | 21 | 0.2 |
| 1a22 | A | Q | 22 | -0.2 |
| 1a22 | A | F | 25 | -0.4 |
| 1a22 | A | Y | 42 | 0.2 |
| 1a22 | A | Q | 46 | 0.1 |
| 1a22 | A | S | 51 | 0.3 |
| 1a22 | A | S | 62 | 0.1 |
| 1a22 | A | N | 63 | 0.3 |
| 1a22 | A | E | 65 | -0.5 |
| 1a22 | A | Y | 164 | 0.3 |
| 1a22 | A | R | 167 | 0.3 |
| 1a22 | A | K | 168 | -0.2 |
| 1a22 | A | K | 172 | 2 |
| 1a22 | A | E | 174 | -0.9 |
| 1a22 | A | T | 175 | 2 |
| 1a22 | A | R | 178 | 2.4 |
| 1a22 | B | R | 243 | 2.12 |
| 1a22 | B | T | 273 | 0.11 |
| 1a22 | B | Q | 274 | 0 |
| 1a22 | B | E | 275 | -0.1 |
| 1a22 | B | W | 280 | -0.02 |
| 1a22 | B | S | 298 | -0.05 |
| 1a22 | B | S | 302 | -0.2 |
| 1a22 | B | W | 304 | 4.5 |
| 1a22 | B | E | 320 | -0.19 |
| 1a22 | B | K | 321 | 0.08 |
| 1a22 | B | S | 324 | 0.28 |
| 1a22 | B | I | 365 | 2.13 |
| 1a22 | B | Q | 366 | 0.02 |
| 1a22 | B | K | 367 | -0.02 |
| 1a22 | B | W | 369 | 4.5 |
| 1a22 | B | V | 371 | -0.64 |
| 1a22 | B | R | 417 | 0.28 |
| 1a22 | B | N | 418 | 0.3 |
| 1a22 | B | S | 419 | 0.03 |
| 1ahw | C | Y | 156 | 4 |
| 1ahw | C | T | 167 | 0 |
| 1ahw | C | D | 178 | -0.5 |
| 1ahw | C | V | 198 | -0.3 |
| 1brs | A | K | 27 | 5.4 |
| 1brs | A | R | 59 | 5.2 |
| 1brs | A | E | 60 | -0.2 |
| 1brs | A | E | 73 | 2.8 |
| 1brs | A | R | 87 | 5.5 |
| 1brs | A | H | 102 | 6 |
| 1brs | D | Y | 29 | 3.4 |
| 1brs | D | D | 35 | 4.5 |
| 1brs | D | D | 39 | 7.7 |
| 1bxi | A | N | 24 | 0.14 |
| 1bxi | A | S | 28 | 0.17 |
| 1bxi | A | L | 33 | 3.42 |
| 1bxi | A | V | 34 | 2.58 |
| 1bxi | A | E | 41 | 2.08 |
| 1bxi | A | S | 48 | 0.01 |
| 1bxi | A | S | 50 | 2.19 |
| 1bxi | A | D | 51 | 5.92 |
| 1bxi | A | Y | 55 | 4.63 |
| 1cbw | I | T | 11 | 0.2 |
| 1cbw | I | K | 15 | 2 |
| 1cbw | I | I | 19 | 0.1 |
| 1cbw | I | V | 34 | 0 |
| 1cbw | I | R | 39 | 0.2 |
| 1dan | T | K | 15 | -0.4 |
| 1dan | T | T | 17 | 0.1 |
| 1dan | T | N | 18 | 0.2 |
| 1dan | T | K | 20 | 2.6 |
| 1dan | T | T | 21 | -0.2 |
| 1dan | T | K | 41 | -0.04 |
| 1dan | T | S | 42 | -0.05 |
| 1dan | T | K | 46 | 0.25 |
| 1dan | T | S | 47 | 0.05 |
| 1dan | T | D | 58 | 2.18 |
| 1dan | T | K | 68 | -0.1 |
| 1dvf | A | Y | 32 | 2 |
| 1dvf | A | W | 92 | 0.3 |
| 1dvf | B | W | 52 | 4.2 |
| 1dvf | B | D | 54 | 4.3 |
| 1dvf | B | E | 98 | 4.2 |
| 1dvf | B | D | 100 | 2.8 |
| 1dvf | B | Y | 101 | 4 |
| 1fc2 | C | I | 150 | 2.2 |
| 1fcc | C | T | 25 | 0.24 |
| 1fcc | C | E | 27 | 4.9 |
| 1fcc | C | K | 31 | 3.5 |
| 1fcc | C | N | 35 | 2.4 |
| 1fcc | C | D | 40 | 0.3 |
| 1fcc | C | W | 43 | 3.8 |
| 1gc1 | C | S | 23 | 0.29 |
| 1gc1 | C | Q | 25 | 0.03 |
| 1gc1 | C | H | 27 | 0.28 |
| 1gc1 | C | N | 32 | 0.18 |
| 1gc1 | C | Q | 33 | 0.1 |
| 1gc1 | C | K | 35 | 0.32 |
| 1gc1 | C | Q | 40 | -0.41 |
| 1gc1 | C | S | 42 | 0 |
| 1gc1 | C | T | 45 | -0.15 |
| 1gc1 | C | S | 60 | -0.09 |
| 1gc1 | C | D | 63 | -0.32 |
| 1jrh | L | W | 92 | 2.8 |
| 1jrh | L | T | 94 | 0.36 |
| 1jrh | H | W | 52 | 2.7 |
| 1jrh | H | W | 53 | 2.4 |
| 1jrh | I | K | 47 | 3.6 |
| 1jrh | I | Y | 49 | 3.4 |
| 1jrh | I | K | 52 | 3 |
| 1jrh | I | N | 53 | 3.9 |
| 1jrh | I | S | 54 | 0.3 |
| 1jrh | I | E | 55 | -0.4 |
| 1jrh | I | W | 82 | 4.5 |
| 1jrh | I | R | 84 | -0.3 |
| 1jrh | I | K | 98 | 0 |
| 1vfb | A | T | 53 | -0.23 |
| 1vfb | A | S | 93 | 0.11 |
| 1vfb | B | T | 30 | 0.09 |
| 1vfb | B | D | 100 | 3.1 |
| 1vfb | B | Y | 101 | 4 |
| 1vfb | C | D | 18 | 0.3 |
| 1vfb | C | N | 19 | 0.3 |
| 1vfb | C | Q | 121 | 2.9 |
| 1vfb | C | L | 129 | 0.2 |
| 2ptc | I | K | 15 | 10 |
| 3hfm | H | S | 31 | 0.2 |
| 3hfm | H | D | 32 | 2 |
| 3hfm | H | Y | 33 | 6 |
| 3hfm | H | Y | 50 | 7.5 |
| 3hfm | H | Y | 53 | 3.29 |
| 3hfm | Y | Y | 20 | 5 |
| 3hfm | Y | W | 63 | 0.3 |
| 3hfm | Y | R | 73 | -0.2 |
| 3hfm | Y | T | 89 | 0 |
| 3hfm | Y | K | 96 | 7 |
| 3hfm | Y | K | 97 | 6 |
| 3hfm | Y | S | 100 | 0.25 |
| 3hfm | Y | H | 15 | -0.5 |
| 3hfm | L | N | 31 | 5.25 |
| 3hfm | L | N | 32 | 5.2 |
| 3hfm | L | Y | 50 | 4.6 |
| 3hfm | L | Y | 96 | 2.8 |
