# Supplementary material for: APIS: accurate prediction of hot spots in protein interfaces by combining protrusion index with solvent accessibility
Source: BMC Bioinformatics. 2010 Apr 8;11:174. doi: 10.1186/1471-2105-11-174 (PMC2874803; doi:10.1186/1471-2105-11-174)
Supplement: Additional file 2 — Summary of the features used in this study. These features can be roughly divided into three groups: (i) physicochemical features; (i) features based on protein tertiary structures; and (iii) residue-residue pairing preferences at the interface, residue evolutionary conservation scores and temperature factors. [file 1471-2105-11-174-S2.DOC]

Table S2. Summary of the features used in this study.

| No. | Feature description | Symbol |
| --- | --- | --- |
| 1 | Number of atoms | Na |
| 2 | Number of electrostatic charge | Nec |
| 3 | Number of potential hydrogen bonds | Nphb |
| 4 | Hydrophobicity | Hdrpo |
| 5 | Hydrophilicity | Hdrpi |
| 6 | Propensity | Prop |
| 7 | Isoelectric point | Isoep |
| 8 | Mass | Mass |
| 9 | Expected number of contacts within 14 Å sphere | Enc |
| 10 | Electron-ion interaction potential | Eiip |
| 11 | Bound total ASA | BtASA |
| 12 | Bound backbone ASA | BbASA |
| 13 | Bound side-chain ASA | BsASA |
| 14 | Bound polar ASA | BpASA |
| 15 | Bound non-polar ASA | BnASA |
| 16 | Bound total RASA | BtRASA |
| 17 | Bound backbone RASA | BbRASA |
| 18 | Bound side-chain RASA | BsRASA |
| 19 | Bound polar RASA | BpRASA |
| 20 | Bound non-polar RASA | BnRASA |
| 21 | Bound total mean DI | BtmDI |
| 22 | Bound side-chain mean DI | BsmDI |
| 23 | Bound maximum DI | BmaxDI |
| 24 | Bound minimal DI | BminDI |
| 25 | Bound total mean PI | BtmPI |
| 26 | Bound side-chain mean PI | BsmPI |
| 27 | Bound maximum PI | BmaxPI |
| 28 | Bound minimal PI | BminPI |
| 29 | Unbound total ASA | UtASA |
| 30 | Unbound backbone ASA | UbASA |
| 31 | Unbound side-chain ASA | UsASA |
| 32 | Unbound polar ASA | UpASA |
| 33 | Unbound non-polar ASA | UnASA |
| 34 | Unbound total RASA | UtRASA |
| 35 | Unbound backbone RASA | UbRASA |
| 36 | Unbound side-chain RASA | UsRASA |
| 37 | Unbound polar RASA | UpRASA |
| 38 | Unbound non-polar RASA | UnRASA |
| 39 | Unbound total mean DI | UtmDI |
| 40 | Unbound side-chain mean DI | UsmDI |
| 41 | Unbound maximum DI | UmaxDI |
| 42 | Unbound minimal DI | UminDI |
| 43 | Unbound total mean PI | UtmPI |
| 44 | Unbound side-chain mean PI | UsmPI |
| 45 | Unbound maximum PI | UmaxPI |
| 46 | Unbound minimal PI | UminPI |
| 47 | Relative change in total ASA upon complexation | RctASA |
| 48 | Relative change in backbone ASA upon complexation | RcbASA |
| 49 | Relative change in side-chain ASA upon complexation | RcsASA |
| 50 | Relative change in polar ASA upon complexation | RcpASA |
| 51 | Relative change in non-polar ASA upon complexation | RcnASA |
| 52 | Relative change in total mean DI upon complexation | RctmDI |
| 53 | Relative change in side-chain mean DI upon complexation | RcsmDI |
| 54 | Relative change in maximum DI upon complexation | RcmaxDI |
| 55 | Relative change in minimal DI upon complexation | RcminDI |
| 56 | Relative change in total mean PI upon complexation | RctmPI |
| 57 | Relative change in side-chain mean PI upon complexation | RcsmPI |
| 58 | Relative change in maximum PI upon complexation | RcmaxPI |
| 59 | Relative change in minimal PI upon complexation | RcminPI |
| 60 | Pairwise potential | Pp |
| 61 | Temperature factor | Tf |
| 62 | Residue conservation | Rc |
