# Supplementary material for: APIS: accurate prediction of hot spots in protein interfaces by combining protrusion index with solvent accessibility
Source: BMC Bioinformatics. 2010 Apr 8;11:174. doi: 10.1186/1471-2105-11-174 (PMC2874803; doi:10.1186/1471-2105-11-174)
Supplement: Additional file 4 — The average prediction results of multi-property SVMs for different number of properties based on independent test set. The feature RcsASA, RctASA, RcpASA, BsRASA, RcsmPI, BtRASA, BpRASA, RctmPI and BsASA were added one by one to construct a series of multi-property SVMs according to the corresponding F-scores. [file 1471-2105-11-174-S4.DOC]

Table S4 The average prediction results of multi-property SVMs for different number of properties based on independent test set. The feature RcsASA, RctASA, RcpASA, BsRASA, RcsmPI, BtRASA, BpRASA, RctmPI and BsASA were added one by one to construct a series of multi-property SVMs according to the corresponding F-scores.

| **Property number** | **Specificity** | **Recall** | **Precision** | **Accuracy** | **F1** | **TP** | **TN** | **FP** | **FN** |
| --- | --- | --- | --- | --- | --- | --- | --- | --- | --- |
| 2 | 0.72 | 0.64 | 0.50 | 0.69 | 0.56 | 25 | 63 | 25 | 14 |
| 3 | 0.68 | 0.72 | 0.50 | 0.69 | 0.59 | 28 | 60 | 28 | 11 |
| 4 | 0.69 | 0.69 | 0.50 | 0.69 | 0.58 | 27 | 61 | 27 | 12 |
| 5 | 0.73 | 0.69 | 0.53 | 0.72 | 0.60 | 27 | 64 | 24 | 12 |
| 6 | 0.75 | 0.67 | 0.54 | 0.72 | 0.60 | 26 | 66 | 22 | 13 |
| 7 | 0.73 | 0.69 | 0.53 | 0.72 | 0.60 | 27 | 64 | 24 | 12 |
| 8 | 0.73 | 0.64 | 0.51 | 0.70 | 0.57 | 25 | 64 | 24 | 14 |
| 9 | 0.73 | 0.67 | 0.52 | 0.71 | 0.58 | 26 | 64 | 24 | 13 |
