# Supplementary material for: APIS: accurate prediction of hot spots in protein interfaces by combining protrusion index with solvent accessibility
Source: BMC Bioinformatics. 2010 Apr 8;11:174. doi: 10.1186/1471-2105-11-174 (PMC2874803; doi:10.1186/1471-2105-11-174)
Supplement: Additional file 5 — Comparison of methodologies. The methodological difference between our method and the other two previous methods of Tuncbag et al. (2009) and Cho et al. (2009). [file 1471-2105-11-174-S5.DOC]

Table S5 The methodological difference between our method and the other two previous methods of Tuncbag *et al.* (2009) and Cho *et al.* (2009).

| Difference | Our method (APIS) | Tuncbag’s method | Cho’s method |
| --- | --- | --- | --- |
| Number of initial features | 62 | 5 | 54 |
| Number of final features | 9 | 2 | 12 |
| Feature selection | Yes (F-score method) | No | Yes (Decision tree method) |
| Same Features used | Accessibility, Residue Conservation, Pair Poteintials | Accessibility, Residue Conservation, Pair Poteintials, | Accessibility, Residue Conservation |
| Different Features used | Physicochemical features (including number of atoms, number of electrostatic charge, number of potential hydrogen bonds, hydrophobicity, hydrophilicity, propensity, isoelectric point, mass, expected number of contacts within 14 Å sphere, and electron-ion interaction potential), Temperature factor, Depth index (DI), Protrusion index (PI) | Computational Alanine Scanning (Robetta) | Residue contact, Atom contact, Weighted density, Molecular interaction information (include molecular interactions of an interface residue and molecular interactions within a residue’s microenvironment) |
| Side-chain information | Yes, (Protein structural information based on the side-chain information, such as Bound side-chain ASA, Bound side-chain mean DI, Relative change in side-chain mean DI upon complexation, Bound side-chain mean PI and so on) | No | No |
| ASA/RASA based on different atom values | In addition to the total values (the sum of all atom values), we also used the backbone (sum of all backbone atom values), side-chain (sum of all side-chain atom values), polar (sum of all oxygen, nitrogen atom values) and non-polar (sum of all carbon atom values) values. | Only the total values (the sum of all atom values) | Only the total values (the sum of all atom values) |
| Classifier | SVM | an intuitive rule | SVM |
| Ensemble method | Yes (majority voting) | No | No |
| F1 score | 0.72 (based on the Tuncbag’s dataset derived from BID),  0.64 (based on the Cho’s dataset derived from BID) | 0.65 (based on the Tuncbag’s dataset derived from BID) | 0.52 (based on the Cho’s dataset derived from BID) |
