# Supplementary material for: APIS: accurate prediction of hot spots in protein interfaces by combining protrusion index with solvent accessibility
Source: BMC Bioinformatics. 2010 Apr 8;11:174. doi: 10.1186/1471-2105-11-174 (PMC2874803; doi:10.1186/1471-2105-11-174)
Supplement: Additional file 6 — Performance based on the Tuncbag et al. dataset assembled from BID. Table S6: Performance of individual-feature based SVM models; Table S7: Evaluation of hot spot prediction using the majority voting method; Table S8: Performance comparison with different methods. [file 1471-2105-11-174-S6.DOC]

**Performance based on the Tuncbag *et al.* dataset assembled from BID** Table S6 Performance of individual-feature based SVM models.

| **Feature** | **Specificity** | **Recall** | **Precision** | **Accuracy** | **F1** | **TP** | **TN** | **FP** | **FN** |
| --- | --- | --- | --- | --- | --- | --- | --- | --- | --- |
| RcsASA | 0.62 | 0.65 | 0.61 | 0.63 | 0.63 | 35 | 36 | 22 | 19 |
| RctASA | 0.71 | 0.63 | 0.67 | 0.67 | 0.65 | 34 | 41 | 17 | 20 |
| RcpASA | 0.71 | 0.48 | 0.60 | 0.60 | 0.54 | 26 | 41 | 17 | 28 |
| BsRASA | 0.05 | 0.78 | 0.43 | 0.40 | 0.56 | 42 | 3 | 55 | 12 |
| RcsmPI | 0.60 | 0.74 | 0.63 | 0.67 | 0.68 | 40 | 35 | 23 | 14 |
| BtRASA | 0.03 | 0.76 | 0.42 | 0.38 | 0.54 | 41 | 2 | 56 | 13 |
| BpRASA | 0.16 | 0.85 | 0.48 | 0.49 | 0.62 | 46 | 9 | 49 | 8 |
| RctmPI | 0.55 | 0.67 | 0.58 | 0.61 | 0.62 | 36 | 32 | 26 | 18 |
| BsASA | 0.74 | 0.50 | 0.64 | 0.63 | 0.56 | 27 | 43 | 15 | 27 |

Table S7 Evaluation of hot spot prediction using the majority voting method.

| **Classifier number** | **Specificity** | **Recall** | **Precision** | **Accuracy** | **F1** | **TP** | **TN** | **FP** | **FN** |
| --- | --- | --- | --- | --- | --- | --- | --- | --- | --- |
| 9 | 0.52 | 0.80 | 0.61 | 0.65 | 0.69 | 43 | 30 | 28 | 11 |
| 7(F1>0.54) | 0.55 | 0.81 | 0.63 | 0.68 | 0.71 | 44 | 32 | 26 | 10 |
| 3(F1>0.62) | 0.60 | 0.80 | 0.65 | 0.70 | 0.72 | 43 | 35 | 23 | 11 |

Table S8 Performance comparison with different methods.

| **Method** | **Recall** | **Precision** | **F1** |
| --- | --- | --- | --- |
| Robetta | 0.57 | 0.63 | 0.60 |
| KFC | 0.36 | 0.51 | 0.42 |
| KFCA | 0.48 | 0.53 | 0.51 |
| LDA | 0.57 | 0.72 | 0.64 |
| Tuncbag’s method | 0.59 | **0.73** | 0.65 |
| APIS (this work) | **0.80** | 0.65 | **0.72** |

The highest value in each column is shown in bold.
