# Supplementary material for: APIS: accurate prediction of hot spots in protein interfaces by combining protrusion index with solvent accessibility
Source: BMC Bioinformatics. 2010 Apr 8;11:174. doi: 10.1186/1471-2105-11-174 (PMC2874803; doi:10.1186/1471-2105-11-174)
Supplement: Additional file 7 — Performance on the test set (BID). Detailed prediction results for the protein structures obtained with our method. [file 1471-2105-11-174-S7.DOC]

Table S9 Detailed prediction results for the structures in the test set (BID).

| **Interface** | **Chain** | **Residue** | **Strength^1^** | **Observed^2^** | **KFC** | **MINERVA** | **APIS** |
| --- | --- | --- | --- | --- | --- | --- | --- |
| 1cdlAE | A | F12 | N | -- | -- | ** | -- |
| 1cdlAE | A | F19 | W | -- | ** | -- | ** |
| 1cdlAE | A | F92 | S | ** | ** | ** | ** |
| 1cdlAE | E | K799 | N | -- | -- | -- | -- |
| 1cdlAE | E | W800 | S | ** | ** | ** | ** |
| 1cdlAE | E | K802 | I | -- | -- | -- | ** |
| 1cdlAE | E | G804 | S | ** | -- | -- | ** |
| 1cdlAE | E | R808 | I | -- | ** | -- | ** |
| 1cdlAE | E | I810 | S | ** | ** | ** | ** |
| 1cdlAE | E | G811 | I | -- | -- | -- | -- |
| 1cdlAE | E | R812 | S | ** | -- | ** | ** |
| 1cdlAE | E | L813 | S | ** | ** | -- | ** |
| 1dvaHX | H | G38 | I | -- | -- | -- | ** |
| 1dvaHX | H | I65 | I | -- | -- | -- | -- |
| 1dvaHX | H | V67 | I | -- | -- | -- | ** |
| 1dvaHX | H | E70 | W | -- | -- | -- | ** |
| 1dvaHX | H | L73 | I | -- | ** | ** | ** |
| 1dvaHX | H | S74 | I | -- | -- | -- | -- |
| 1dvaHX | H | E75 | I | -- | -- | -- | -- |
| 1dvaHX | H | H76 | S | ** | -- | -- | -- |
| 1dvaHX | H | E80 | I | -- | -- | -- | -- |
| 1dvaHX | H | S82 | I | -- | -- | -- | -- |
| 1dvaHX | H | L144 | I | -- | -- | -- | -- |
| 1dvaHX | H | L153. | W | -- | -- | -- | -- |
| 1dvaHX | X | A1 | I | -- | -- | -- | -- |
| 1dvaHX | X | L2 | S | ** | -- | ** | ** |
| 1dvaHX | X | D5 | W | -- | -- | -- | -- |
| 1dvaHX | X | R7 | W | -- | -- | -- | -- |
| 1dvaHX | X | V8 | I | -- | -- | -- | -- |
| 1dvaHX | X | D9 | I | -- | -- | ** | -- |
| 1dvaHX | X | W11 | S | ** | -- | -- | ** |
| 1dvaHX | X | Y12 | S | -- | ** | -- | ** |
| 1dvaHX | X | Q14 | I | -- | -- | -- | -- |
| 1dvaHX | X | F15 | S | ** | ** | ** | ** |
| 1dvaHX | X | V16 | I | -- | -- | -- | -- |
| 1dx5BN | N | I24 | I | -- | -- | -- | -- |
| 1dx5BN | N | K235 | I | -- | -- | -- | -- |
| 1dx5BJ | N | F34 | I | -- | ** | -- | -- |
| 1dx5BJ | N | K36 | W | -- | -- | -- | -- |
| 1dx5BJ | N | P37 | W | -- | -- | -- | -- |
| 1dx5BJ | N | Q38 | W | -- | -- | ** | ** |
| 1dx5BJ | N | E39 | I | -- | -- | -- | -- |
| 1dx5BJ | N | L65 | W | -- | -- | -- | -- |
| 1dx5BJ | N | R67 | S | ** | -- | -- | ** |
| 1dx5BJ | N | T74 | W | -- | -- | -- | -- |
| 1dx5BJ | N | R75 | W | -- | -- | -- | -- |
| 1dx5BJ | N | Y76 | S | ** | ** | -- | ** |
| 1dx5BJ | N | E80 | S | ** | -- | -- | -- |
| 1dx5BJ | N | K81 | W | -- | -- | -- | -- |
| 1dx5BJ | N | I82 | I | -- | -- | ** | ** |
| 1dx5BJ | N | M84 | I | -- | -- | -- | -- |
| 1dx5BJ | N | K110 | I | -- | -- | -- | -- |
| 1ebpAC | A | F93 | S | ** | -- | -- | -- |
| 1ebpAC | A | M150 | S | ** | -- | ** | ** |
| 1ebpAC | A | T151 | W | -- | -- | -- | -- |
| 1ebpAC | A | F205 | S | ** | -- | -- | -- |
| 1ebpAC | C | G9 | I | -- | -- | -- | -- |
| 1ebpAC | C | P10 | I | -- | -- | -- | -- |
| 1ebpAC | C | L11 | I | -- | -- | -- | -- |
| 1ebpAC | C | T12 | W | -- | -- | -- | -- |
| 1ebpAC | C | W13 | S | ** | -- | ** | ** |
| 1es7AB | A | F49 | I | -- | ** | ** | ** |
| 1es7AB | A | P50 | I | -- | ** | ** | ** |
| 1es7AC | A | V26 | I | -- | -- | -- | ** |
| 1es7AD | A | W31 | S | ** | -- | ** | -- |
| 1fakHT | T | Q37 | W | -- | ** | -- | -- |
| 1fakHT | T | K41 | I | -- | -- | -- | -- |
| 1fakHT | T | S42 | I | -- | -- | -- | -- |
| 1fakHT | T | D44 | W | -- | -- | -- | -- |
| 1fakHT | T | Y94 | W | -- | -- | -- | -- |
| 1fakLT | T | K15 | I | -- | -- | -- | -- |
| 1fakLT | T | T17 | I | -- | -- | -- | -- |
| 1fakLT | T | N18 | I | -- | -- | -- | ** |
| 1fakLT | T | K20 | S | ** | -- | ** | ** |
| 1fakLT | T | I22 | W | -- | -- | -- | -- |
| 1fakLT | T | E24 | W | -- | -- | -- | -- |
| 1fakLT | T | S47 | I | -- | -- | -- | -- |
| 1fakLT | T | K48 | I | -- | ** | -- | -- |
| 1fakLT | T | F50 | I | -- | -- | -- | -- |
| 1fakLT | T | D58 | S | ** | -- | ** | ** |
| 1fakLT | T | E128 | I | -- | -- | -- | -- |
| 1fakLT | T | L133 | I | -- | -- | -- | ** |
| 1fakLT | T | R135 | I | -- | -- | -- | -- |
| 1fakLT | T | F140 | I | -- | -- | -- | -- |
| 1fakLT | T | T203 | I | -- | ** | -- | -- |
| 1fakLT | T | V207 | I | -- | -- | -- | -- |
| 1fe8AH | A | E987 | I | -- | -- | -- | ** |
| 1fe8AH | A | H990 | I | -- | ** | -- | ** |
| 1fe8AL | A | R963 | I | -- | -- | -- | -- |
| 1fe8AL | A | E987 | I | -- | -- | -- | ** |
| 1fe8AL | A | H1023 | I | -- | -- | -- | -- |
| 1foeAB | B | S41 | I | -- | -- | -- | -- |
| 1foeAB | B | G54 | S | ** | -- | -- | ** |
| 1g3iAG | A | D438 | S | ** | -- | -- | -- |
| 1g3iAG | A | L439 | S | ** | -- | -- | -- |
| 1g3iAG | A | R441 | S | ** | -- | -- | ** |
| 1g3iAG | A | F442 | S | ** | -- | ** | ** |
| 1g3iAG | A | I443 | S | ** | -- | ** | ** |
| 1g3iAG | A | L444 | S | ** | -- | ** | ** |
| 1gl4AB | A | R403 | I | -- | -- | -- | -- |
| 1gl4AB | A | D427 | S | ** | -- | -- | -- |
| 1gl4AB | A | H429 | S | ** | -- | ** | ** |
| 1gl4AB | A | Y431 | S | ** | ** | -- | ** |
| 1gl4AB | A | Y440 | I | -- | ** | ** | ** |
| 1gl4AB | A | E616 | S | ** | ** | -- | -- |
| 1gl4AB | A | R620 | S | ** | ** | ** | ** |
| 1ihbAB | B | N101 | I | -- | -- | -- | -- |
| 1ihbAB | B | R133 | W | -- | -- | -- | -- |
| 1ihbAB | B | H135 | W | -- | -- | -- | -- |
| 1ihbAB | B | K136 | I | -- | -- | -- | -- |
| 1jatAB | A | E55 | S | ** | -- | -- | ** |
| 1jatAB | B | F8 | S | ** | ** | ** | ** |
| 1jppBD | B | K345 | S | ** | -- | -- | -- |
| 1jppBD | B | K354 | I | -- | -- | -- | -- |
| 1jppBD | B | W383 | S | ** | -- | -- | -- |
| 1jppBD | B | R386 | I | -- | -- | -- | -- |
| 1jppBD | B | K435 | I | -- | -- | -- | -- |
| 1jppBD | B | R469 | I | -- | -- | -- | -- |
| 1jppBD | B | H470 | I | -- | -- | -- | -- |
| 1mq8AB | B | T206 | S | ** | ** | -- | ** |
| 1nfiAF | F | C215 | I | -- | -- | -- | ** |
| 1nfiBF | F | Y181 | S | ** | -- | ** | ** |
| 1nunAB | A | D76 | I | -- | ** | -- | ** |
| 1nunAB | A | R78 | I | -- | -- | -- | ** |
| 1nunAB | A | R155 | I | -- | ** | -- | -- |
| 1ub4AC | C | F453 | I | -- | -- | -- | -- |
| 2hhbAB | B | Y35 | I | -- | -- | -- | -- |

**^1^**Interaction strength: W (weak), I (intermediate), N (insignificant), S (strong).

**^2^****represents hot spot, -- represents non-hot spot.
